# Evaluation of the functional performance and technical quality of an Electronic Documentation System of the Nursing Process^1^

**DOI:** 10.1590/0104-1169.3562.2548

**Published:** 2015-04-14

**Authors:** Neurilene Batista de Oliveira, Heloisa Helena Ciqueto Peres

**Affiliations:** 2 MSc, RN, Hospital Universitário, Universidade de São Paulo, São Paulo, SP, Brazil; 3 PhD, Full Professor, Escola de Enfermagem, Universidade de São Paulo, São Paulo, SP, Brazil

**Keywords:** Nursing Informatics, Software, Technology Assessment, Biomedical, Nursing Diagnosis

## Abstract

**Objective:**

To evaluate the functional performance and the technical quality of the Electronic Documentation System of the Nursing Process of the Teaching Hospital of the University of São Paulo.

**Method:**

exploratory-descriptive study. The Quality Model of regulatory standard 25010 and the Evaluation Process defined under regulatory standard 25040, both of the International Organization for Standardization/International Electrotechnical Commission. The quality characteristics evaluated were: functional suitability, reliability, usability, performance efficiency, compatibility, security, maintainability and portability. The sample was made up of 37 evaluators.

**Results:**

in the evaluation of the specialists in information technology, only the characteristic of usability obtained a rate of positive responses of less than 70%. For the nurse lecturers, all the quality characteristics obtained a rate of positive responses of over 70%. The staff nurses of the medical and surgical clinics with experience in using the system) and staff nurses from other units of the hospital and from other health institutions (without experience in using the system) obtained rates of positive responses of more than 70% referent to the functional suitability, usability, and security. However, performance efficiency, reliability and compatibility all obtained rates below the parameter established.

**Conclusion:**

the software achieved rates of positive responses of over 70% for the majority of the quality characteristics evaluated.

## Introduction

The use of computing systems and software has grown rapidly in all sectors of activity of society, reaching an ever greater number of users. Software is used in the areas of education, entertainment, transport, communication, the financial system, the environment, industry, business, medicine and many others. 

The quality of the health information systems is a worldwide concern, and a series of initiatives is being undertaken in countries including the United States, Canada and the United Kingdom, so as to promote security in the design, acquisition and implantation of information technology in the health area^(^ 1 ^)^.

In Brazil, the implantation of computational tools integrated with the Electronic Health Record (EHR) for documenting the Nursing Process (NP) has been a gradual process, and is found in different stages of implementation.

In this context, the Teaching Hospital of the University of São Paulo (HU-USP), in partnership with the University of São Paulo's School of Nursing (EEUSP) developed the University of São Paulo Nursing Process Electronic Documentation System (PROCEnf-USP^(r))^, using the NANDA-I (NANDA - International), NIC (Nursing Interventions Classification) and NOC (Nursing Outcomes Classification)^(^ 2 ^)^.

It is considered that the development of this software represents an advance for nursing through its use of standardized systems of language which serve to demonstrate nursing's contribution to individuals' health, allowing the measuring both of clinical efficacy and the cost of the nursing care. 

Nevertheless, ensuring this system's quality is an important challenge and goal due to the responsibility for users and patients. It is believed that a new technology can bring profound transformations, and that a system can be considered successful when it meets the users' needs, is easy to use, is not prone to failure, and changes things for the better^(^ 3 ^)^.

The PROCEnf-USP^(r)^ was evaluated during the implementation phase, and the nurses judged items such as visual comfort and the handling of the system, documentation, information and content. The nurses evaluated the system positively, mainly because it contributes to the nurse's clinical reasoning and because it relates diagnoses, results and interventions^(^ 4 ^)^.

In the international scenario, the experience of administrators, nursing managers, nurses and other users with the implementation of EHR evidenced the achievement of operational advances through greater access to information, increasing the accuracy of documentation, implementation of evidence-based practice and cost reduction, as well as improvements in the quality of the care and greater staff satisfaction. Emphasis is also placed on the positive evaluation from the administrators in relation to the return on the investments^(^ 5 ^)^.

One study on the evaluation of changes in the quality of the processing of information in nursing, immediately prior to, and one year after, the introduction of a computerized nursing system concluded that there had been significant improvement in the quality of the nursing documentation, as well as support during the patient anamnesis and care planning. The authors observed a lack of clarity in relation to the time spent on the electronic nursing documentation and its impact on patient care^(^ 6 ^)^.

One of the approaches for evaluating information technology is the use of quality standards devised and revised by the ISO (International Organization for Standardization) and the IEC (International Electrotechnical Commission). The ABNT (Brazilian Association of Technical Standards) series - Brazilian Regulatory Standard (NBR) ISO/IEC 9126^(^ 7 ^)^ and ISO/IEC 14598^(^ 8 ^)^ deal with the quality of software products. In 2011, these regulatory standards were restructured and came to be termed ISO/IEC 25010 - System and Software engineering - (SQuaRE) - System and software quality models^(^ 9 ^)^ and ISO/IEC 25040 System and Software engineering - (SQuaRE) - Evaluation process^(^ 10 ^)^.

In Brazil, these norms were used for evaluating the technical quality and functional performance of a prototype-software for documenting the NP, contributing to identifying the quality of the system and elucidating the needs for improvements so as to optimize the use of the tool^(^ 3 ^)^.

In the light of the above, it was considered fundamental to undertake an evaluation of the PROCEnf-USP(r) system following its implantation, with a view to measuring the performance of the system and the user satisfaction. A further aim was to identify possible technical shortcomings and limitations such that improvements could be made in the final product.

As a result, this study aimed to evaluate the functional performance and technical quality of the PROCEnf-USP(r) system, using the ISO/IEC 25010 Product Quality Model^(^ 9 ^)^ and the ISO/IEC 25040 Evaluation Process^(^ 10 ^)^.

## Method

The PROCEnf-USP^(r)^ system was implanted in 2009, in the General Medicine and Surgical units of the HU-USP. These two clinics were chosen as they are inpatient treatment units for adult patients with strongly consolidated NP, it being possible to replicate the results of the implantation in the hospital's other units. 

The main characteristics of the PROCEnf-USP^(r)^ relate to the possibility of access to two environments: the professional and the academic. The professional environment is exclusively for use in the hospital for real clinical documentation. The academic environment can be accessed both from the hospital and from the EEUSP, and allows the creation of fictitious patients for promoting learning through the simulation of teaching situations with the same characteristics as real documentation. It also stands out because it is characterized as a system for supporting decisions which assists the nurse's clinical reasoning as it has the capacity to generate suggestions for diagnoses, results, and nursing interventions based on the data from the evaluation^(^ 2 ^)^.

This is an exploratory descriptive study. The sample was intentional and non-probabilistic, made up of 37 (thirty seven) evaluators, of whom eight (8) were specialists in information technology, eight (8) were nurse lecturers, thirteen (13) were staff nurses from the general medicine and surgical clinics (with experience in the use of the PROCEnf-USP^(r) ^system) and eight (8) were staff nurses from other units of the HU-USP and from other health institutions (without experience in the use of the above-mentioned system).

The inclusion criteria were: specialists in information technology a) to have, at the minimum, the title of specialist in information technology; and b) to have professional experience in systems analysis. Nurse lecturers: a) to have, at the minimum, the title of specialist in nursing; b) to be a lecturer in an institute of higher education which teaches nursing; and c) to run courses or participate in study groups involving the NP. Staff nurses from the medical and surgical clinics: a) to be a nurse working in the care area; b) to have, at the minimum, the title of specialist in nursing; c) to use the PROCEnf-USP^(r)^ system in the care, d) to have experience in the use of the NP. Staff nurses from other units: a) to be a staff nurse of the units of the HU-USP which do not use the PROCEnf-USP^(r)^ system in the care, or to be a staff nurse from another health institution; b) to have, at the minimum, the title of specialist in nursing; and c) to have experience in the use of the NP. 

The first stage of the Evaluation Process was the choice of the ISO/IEC 25010 Product Quality Model^(^ 9 ^)^, which specifies eight quality characteristics, subdivided into sub-characteristics, as follows: functional suitability (functional completeness, functional correctness, and functional appropriateness); performance efficiency (time-behavior, resource utilization and capacity); compatibility (co-existence and interoperability); usability (appropriateness recognizability, learnability, user error protection, operability, user interface aesthetics and accessibility); reliability (maturity, fault tolerance, recoverability and availability); security (confidentiality, integrity, non-repudiation, accountability and authenticity); maintainability: (analyzability, modifiability, modularity, reusability, and testability); portability (adaptability, installability and replaceability). These two last characteristics were evaluated only by the specialists in information technology. 

The quality characteristics and sub-characteristics were evaluated through key questions, adapted from the evaluation instrument used by Sperandio^(^ 3 ^)^. Following the updating of the standard, new characteristics were added and others received more accurate names. Due to this change, it was necessary to add new questions to the evaluation instruments. For this, the SBIS/CFM Certification Manual was used, principally for the questions regarding the characteristic of security. 

In the second stage of the evaluation, the scoring levels and the criteria for judgment were defined. For each question from the evaluation instruments, the specialists attributed the following scores: A (Agree); D (Disagree), NA (Not Applicable). Score A signifies that the PROCEnf-USP^(r)^ meets the requirement; D, that it does not meet the requirement, and NA corresponds to the attribute that it was either not evaluated or was considered not to be applicable to the software. Those items evaluated as Disagree were explained in writing by the evaluators, so as to identify the needs for improvement in the PROCEnf-USP^(r)^.

In order to obtain the values of the quality characteristics, following application of the evaluation instruments, the formula for calculating the characteristics and sub-characteristics proposed in ABNT NBR ISO/IEC 14598-6 Annex C (Informational)^(^ 11 ^)^ was adapted: Vc = ∑ Vsc/nsc and Vsc = ∑ m/ (n-nd). In which: Vc is the value of the characteristic measured; Vsc is the value of the sub-characteristic measured; nsc is the number of sub-characteristics; m is 1, if the response is positive, otherwise being 0; n is the total number of measurements; nd is the number of questions discarded. 

Based in this formula for calculating characteristics, the 'Not Applicable' responses were discarded, as the specialists were not able to evaluate these, either as a result of lack of resources, lack of information, or even lack of specific knowledge on the subject addressed. This type of response is not scored and cannot impair the evaluation of the product. 

The tabulation of the data and the calculation of the characteristics were undertaken using the Excel^(r)^ tool. The criteria for judging the results obtained were based in the evaluation scale for sub-characteristics proposed in ABNT NBR ISO/IEC 14598-6 Annex C (Informational)^(^ 11 ^)^, adapted by Sperandio^(^ 3 ^)^, which indicates the values expected for each one of the characteristics and sub-characteristics. The value expected was that each characteristic evaluated would obtain a rate of positive responses of over 70%. 

In the third stage, the evaluation plan was produced. The evaluators were invited to participate in the study through a Letter of Invitation, sent by email. After the signing of the terms of consent, instructions was sent for undertaking the evaluation, along with a link for accessing the PROCEnf-USP^(r)^ system over the Internet, an evaluator's password for exclusive access to the system's academic environment, and the registration of a fictitious patient. With the exception of the nurses from the medical and surgical clinics, who already use the system, the evaluators were sent a manual for use of the PROCEnf-USP^(r) ^system, set up by the management group. Each evaluator received a clinical case, whose use was optional, in order to undertake evaluation of the system. 

The evaluation *per se* was undertaken by the specialists using the Evaluation Instruments sent over the Internet, using the online questionnaire tool SurveyMonkey*(r)*. The evaluators' responses were obtained automatically through the use of this data collection tool. The data collection period was December 2011 - July 2012. This time period was necessary for obtaining the minimum number of eight evaluators for each category, as recommended by ABNT NBR ISO/IEC 14598-6^(^ 11 ^)^.

In the fourth stage, the measurements were obtained, and comparison was undertaken with the criteria, and results were judged. In the fifth stage, the results of the evaluation were analyzed, the conclusion being the declaration of the PROCEnf-USP^(r) ^system's quality.

The project was approved in 2011 in the Research Ethics Committees (REC) of the EEUSP (proposing institution) and of the HU-USP (co-participant institution), with the respective record numbers: REC N. 1031/2011, and REC N. 1132/11, SISNEP CAAE: 0037.0.196.196.11.

## Results

Table 1 shows that, in the evaluation of the specialists in information technology, all the characteristics obtained rates of responses of 'Agree' of below 70%. Maintainability and portability obtained rates of responses of Not Applicable of above 50%. Usability had the highest frequency of responses of 'Disagree'.

**Table 1 -:** Distribution of the frequencies of the responses relating to the evaluation of the quality characteristics of the PROCENF-USP(r) by the specialists in information technology (N=8), São Paulo, State of São Paulo (SP), Brazil, 2012

Characteristics	Agree			Disagree			N/Applicable			Total	
	n	%		n	%		n	%		n	%
Functional Suitability	31	65		3	6		14	29		48	100
Reliability	16	50		5	16		11	34		32	100
Usability	58	56		35	34		11	10		104	100
Performance Efficiency	32	67		6	12		10	21		48	100
Compatibility	12	50		2	8		10	42		24	100
Security	40	56		5	7		27	37		72	100
Maintainability	14	35		1	2		25	63		40	100
Portability	11	46		1	4		12	50		24	100

One can observe in Table 2 that, in the evaluation of the lecturers, the majority of the quality characteristics scored above 70% for responses of 'Agree', apart from compatibility which obtained only 38%. However, it is ascertained that this item obtained 50% for responses of 'Not Applicable'. Usability obtained the highest frequency of responses of 'Disagree'. 

**Table 2 -:** Distribution of the frequencies of the responses relating to the evaluation of the quality characteristics of the PROCENF-USP(r) by the nurse lecturers (N=8), São Paulo, SP, Brazil, 2012

Characteristics	Agree			Disagree			N/Applicable			Total	
	n	%		n	%		n	%		n	%
Functional Suitability	44	92		4	8		0	0		48	100
Reliability	24	75		3	9		5	16		32	100
Usability	83	80		16	15		5	5		104	100
Performance Efficiency	29	73		7	17		4	10		40	100
Compatibility	9	38		3	12		12	50		24	100
Security	45	80		0	0		11	20		56	100

In Table 3, one can observe that, in the evaluation of the staff nurses from the Medical and Surgical Clinics, half of the quality characteristics scored above 70% for responses of 'Agree'. Performance efficiency, reliability and compatibility did not achieve 70% for responses of 'Agree'. There were few responses of 'Not Applicable'. 

**Table 3 -:** Distribution of the frequencies of the responses relating to the evaluation of the quality characteristics of the PROCENF-USP(r) by the staff nurses of the Medical and Surgical Clinics (N=13), São Paulo, SP, Brazil, 2012

Characteristics	Agree			Disagree			N/Applicable			Total	
	n	%		n	%		n	%		n	%
Functional Suitability	63	81		14	18		1	1		78	100
Reliability	35	67		16	31		1	2		52	100
Usability	139	82		25	15		5	3		169	100
Performance Efficiency	30	46		34	52		1	2		65	100
Compatibility	24	62		15	38		0	0		39	100
Security	89	98		2	2		0	0		91	100

According to Table 4, only two quality characteristics obtained over 70% for responses of 'Agree'. Performance efficiency and usability obtained the highest frequency of score for 'Disagree'. Compatibility obtained 45% of responses as 'Not Applicable'. 

**Table 4 -:** Distribution of the frequencies of the responses relating to the evaluation of the quality characteristics of the PROCENF-USP(r) by the staff nurses from Other Units (N=8), São Paulo, SP, Brazil, 2012

Characteristics	Agree		Disagree		N/Applicable		Total	
	f	%	f	%	f	%	f	%
Functional Suitability	36	75	5	10	7	15	48	100
Reliability	14	44	9	28	9	28	32	100
Usability	68	66	17	16	19	18	104	100
Performance Efficiency	17	43	20	50	3	7	40	100
Compatibility	9	38	4	17	11	45	24	100
Security	48	86	0	0	8	14	56	100

In Table 5, as described in the methodology, the responses of 'Not Applicable' were discarded, as they were not applicable to the system or could not be evaluated by the specialists. It is observed that, in the evaluation of the specialists in information technology, only the characteristic of usability obtained rates of positive responses of below 70%. For the nurse lecturers, all the quality characteristics obtained rates of positive responses of above 70%. For the staff nurses from the medical and surgical clinics, and for the staff nurses from other units, functional suitability, usability and security obtained rates of positive responses of over 70%; however, performance efficiency, reliability and compatibility obtained rates below the parameter established. 

**Table 5 -:** Distribution of the responses relating to the quality characteristics of the PROCENF-USP(r) by the specialists in information technology, nurse lecturers, nurses from the medical and surgical clinics and nurses from other units, São Paulo, SP, Brazil, 2012

Characteristics	Specialist in I.T			Nurse Lecturer			Nurse – Medical and Surgical Clinics			Nurse – Other Units	
	Agree	Disagree		Agree	Disagree		Agree	Disagree		Agree	Disagree
Functional Suitability	91%	9%		92%	8%		82%	18%		88%	12%
Reliability	76%	24%		89%	11%		69%	31%		61%	39%
Usability	62%	38%		84%	16%		85%	15%		80%	20%
Performance Efficiency	84%	16%		81%	19%		47%	53%		46%	54%
Compatibility	86%	14%		75%	25%		62%	38%		69%	31%
Security	89%	11%		100%	0%		98%	2%		100%	0%
Maintainability	93%	7%		-	-		-	-		-	-
Portability	92%	8%		-	-		-	-		-	-

## Discussion

The evaluation of the PROCEnf-USP^(r)^ was undertaken by professionals from different areas of knowledge, and the results indicated rates of quality of the system in each attribute considered. It was evidenced that, from the functional point of view, the PROCEnf-USP^(r)^ is a computational tool which meets the users' needs, allows the application of the NP correctly, and facilitates its undertaking. This level of quality is reached when the system's functionalities meet what was stipulated in its requirements. 

It is believed that this success was the result of the participation and involvement of the nursing management, staff nurses, hospital administrators and lecturers of the EEUSP in the development of the PROCEnf-USP^(r)^.

A review of the literature regarding the use of the EHR in nursing demonstrates that the nurses are dissatisfied with the systems, as these were not designed to meet the needs of clinical practice, evaluating the participation of these professionals directly in the development, planning and design of the software as an essential condition if one is to guarantee the complexity and essentiality of the profession^(^ 12 ^)^.

In order to prioritize improvements in the PROCEnf-USP^(r)^, the frequency of the items for which the response was 'Disagree' was analyzed, and it was observed that they were related to the characteristics of usability, performance efficiency, and reliability. 

The nurses' perception regarding the strong points and limitations of the EHRs for documenting clinical events indicates aspects of usability as positive points, and the lack of relevancy of documentation and communication barriers as suggestions for improvements of the system^(^ 13 ^)^.

The evaluation of the usability of the computational tools for the teaching of nursing in intensive care, undertaken by undergraduate students, highlighted positive points in relation to the ease of use and the visualization of the screens. Nevertheless, the item of 'satisfaction with performance in the simulation' did not obtain a satisfactory value, suggesting improvements in the usability^(^ 14 ^)^.

Studies which use methods of heuristic evaluation for the analysis of the electronic nursing records systems, with standardized language, demonstrated that these systems have problems of usability, with negative effects for the nurse's documentation practices and the nursing work^(^ 15 ^)^.

Furthermore, in relation to usability, the specific methods for evaluating effectiveness, efficiency and satisfaction have identified software problems which can lead to operational inefficiencies and errors in coding data, which can bring serious consequences for health^(^ 16 ^)^.

The results of the characteristic of performance efficiency demonstrated that the system's users desire software which is faster in its response time and data processing. The system must allow the nurses to undertake routine tasks with a minimum number of steps. They need to have efficiency mechanisms, avoiding unnecessary rework and facilitating the recovery of data. The explanations provided for the responses of 'Disagree' also evidenced the need for investment in equipment, such as computers and printers. 

In this regard, it is appropriate to consider that the process of implementing and maintaining a computerized system involves costs and requires the directing of concentrated efforts, involving structural, processual and financial variables. Besides this, it requires the management of the nursing department/services to be continuously concerned with how resources are allocated, and with assessing the costs to the institution resulting from these processes. 

The characteristic of reliability requires improvements, as it still presents some shortcomings in the recovery of data affected by failures and freezing of the system. The characteristic of compatibility, particularly in the sub-characteristic of interoperability, obtained a high rate of responses of 'Disagree' in the evaluation of the nurses from the Medical and Surgical Clinics. This evidenced the need for improvements in the PROCEnf-USP^(r)^'s level of communicability with the other hospital systems and in its capacity to transfer data and exchange commands. 

Taking into account that the significant use of electronic records in health has become ubiquitous in the vocabulary of information technology in health, and that information has to flow, interoperability between the systems is essential. It makes the health community more integrated, allowing data to be shared among the various health professionals, the patients and the family, and also allows the health providers to analyze and exchange data between organizations^(^ 17 ^)^.

The characteristics of maintainability and portability reached the necessary percentage in the characteristics evaluated, although a high percentage of responses of 'Not Applicable' was observed. This result was similar to those found in the study undertaken by Sperandio^(^ 3 ^)^ in these two characteristics. 

Compatibility also obtained many responses for 'Not Applicable', except in the evaluation of the nurses from the Medical and Surgical clinics. This type of response is little desirable, as it shows that the resources made available were insufficient for undertaking the evaluation, this being a limitation of this study. 

## Conclusion

The undertaking of this study made it possible to evaluate the technical quality and functional performance of the PROCEnf-USP^(r) ^software, and the results obtained demonstrated the quality of the system in each attribute considered. It is concluded that the PROCEnf-USP(r) system stood out in five quality characteristics: functional suitability, usability, security, maintainability and portability. 

The process of improving the system is continuous, with proposals for implantation in the care units of the hospital, integration with the administrative data from human resources and quality indicators, interoperability with materials systems and patients and tests systems, and the computerized dimensioning of nursing professionals, among others. It is believed that in the near future, standardized electronic documentation will allow remote and simultaneous access to data between health professionals and different organizations. This fact will improve the care provided to the patient and assist in the development of attendance protocols and research. 

In this context, this study will contribute to the improvement of the systems developed for documenting the NP. This study is not terminated in this research report, but opens a path for further studies involving nurses in the evaluation process for software in the area of nursing. 
